# Novel Multidrug-Resistant Enterococcal Mobile Linear Plasmid pELF1 Encoding *vanA* and *vanM* Gene Clusters From a Japanese Vancomycin-Resistant Enterococci Isolate

**DOI:** 10.3389/fmicb.2019.02568

**Published:** 2019-11-13

**Authors:** Yusuke Hashimoto, Makoto Taniguchi, Kazuma Uesaka, Takahiro Nomura, Hidetada Hirakawa, Koichi Tanimoto, Kiyoko Tamai, Genjie Ruan, Bo Zheng, Haruyoshi Tomita

**Affiliations:** ^1^Department of Bacteriology, Gunma University Graduate School of Medicine, Maebashi, Japan; ^2^Oral Microbiome Center, Taniguchi Dental Clinic, Takamatsu, Japan; ^3^Graduate School of Bioagricultural Sciences, Nagoya University, Nagoya, Japan; ^4^Laboratory of Bacterial Drug Resistance, Gunma University Graduate School of Medicine, Maebashi, Japan; ^5^MIROKU Medical Laboratory, Inc., Saku, Japan; ^6^Institute of Clinical Pharmacology, Peking University First Hospital, Beijing, China

**Keywords:** enterococci, linear plasmid, conjugation, vancomycin-resistant enterococci, drug resistance

## Abstract

Vancomycin-resistant enterococci are troublesome pathogens in clinical settings because of few treatment options. A VanA/VanM-type vancomycin-resistant *Enterococcus faecium* clinical isolate was identified in Japan. This strain, named AA708, harbored five plasmids, one of which migrated during agarose gel electrophoresis without S1 nuclease treatment, which is indicative of a linear topology. We named this plasmid pELF1. Whole genome sequencing (WGS) analysis of the AA708 strain revealed that the complete sequence of pELF1 was 143,316 bp long and harbored both the *vanA* and *vanM* gene clusters. Furthermore, mfold analysis and WGS data show that the left end of pELF1 presumably forms a hairpin structure, unlike its right end. The pELF1 plasmid was not digested by lambda exonuclease, indicating that terminal proteins were bound to the 5′ end of the plasmid, similar to the *Streptomyces* linear plasmids. Sodium dodecyl sulfate-polyacrylamide gel electrophoresis results were also consistent with the exonuclease assay results. In retardation assays, DNAs containing the right end of proteinase K-untreated pELF1 did not appear to move as well as the proteinase K-treated pELF1, suggesting that terminal proteins might be attached to the right end of pELF1. Palindromic sequences formed hairpin structures at the right terminal sequence of pELF1; however, sequence similarity with the well-known linear plasmids of *Streptomyces* spp. was not high. pELF1 was unique as it possessed two different terminal structures. Conjugation experiments revealed that pELF1 could be transferred to *E. faecalis*, *E. faecium*, *E. casseliflavus*, and *E. hirae*. These transconjugants exhibited not only high resistance levels to vancomycin, but also resistance to streptomycin, kanamycin, and erythromycin. These results indicate that pELF1 has the ability to confer multidrug resistance to *Enterococcus* spp. simultaneously, which might lead to clinical hazards.

## Introduction

Enterococci, a type of bacteria that are part of the human intestinal microflora, can cause diseases in certain clinical settings. Vancomycin-resistant enterococci, which emerged in the 1980s, have now become important pathogens because of few treatment options. VanA-type and VanB-type VREs are predominant among the nine types of vancomycin resistance. These VanA-type and VanB-type VREs produce a peptidoglycan precursor ending in D-alanyl-D-lactate, which confers a higher level of vancomycin resistance to *Enterococcus* spp. Furthermore, VanM-type vancomycin-resistant bacteria, which also produce the peptidoglycan precursor ending in D-alanyl-D-lactate, was first reported in 2010. In Shanghai, the VanM-type VRE was more prevalent than VanA-type VRE (Xu et al., 2010; Chen et al., 2015). Recently, VREs that harbored both *vanA* and *vanM* gene clusters were identified (Sun H.L. et al., 2019). These double-positive strains exhibited high level resistance to vancomycin. Until now, VanM-type VREs were only isolated in China and Singapore (Teo et al., 2011).

Vancomycin resistance genes, usually encoded on mobile genetic elements, including transposons or plasmids (Werner et al., 2008), are disseminated via conjugative transfer (Arthur and Courvalin, 1993). This plays an important role in the horizontal spread of vancomycin resistance. Among mobile genetic elements, a “plasmid” is originally considered an extrachromosomal genetic element with circular topology (Cornell et al., 2018). In contrast, linear DNA elements have already been reported in various prokaryotes and eukaryotes (Hinnebusch and Tilly, 1993; Cornell et al., 2018). Among them, the first bacterial linear plasmid and linear chromosome was detected in *Streptomyces rochei* and *Borrelia* spp., respectively (Hayakawa et al., 1979; Saint Girons et al., 1994). Recently, linear plasmids were described in *Streptomyces*, *Nocardia*, *Rhodococcus*, *Micrococcus*, *Brevibacterium*, and *Mycobacterium* (Kalkus et al., 1990; Picardeau and Vincent, 1997; Shimizu et al., 2001; Dib et al., 2010, 2013). Plasmids are not unusual in clinical *Enterococcus* strains, and they harbor virulence factors such as genes responsible for resistance to antibiotics, cytolysin, and bacteriocins (Clewell et al., 2014). However, to the best of our knowledge, these plasmids are circular.

In this study, we characterized a multidrug-resistant 143-kb plasmid harboring both the *vanA* and *vanM* gene clusters. The topology of this plasmid was linear, and one of the ends (left end) formed a hairpin-like loop, whereas the other end (right end) is presumed to be of the invertron type (Sakaguchi, 1990; Hinnebusch and Barbour, 1991; Hinnebusch and Tilly, 1993; Dib et al., 2015). In addition, it can be potentially transferred to different enterococcal species and confer high level of vancomycin resistance. Our study shows that this linear plasmid pELF1 may become a significant clinical hazard.

## Materials and Methods

### Strains

The bacterial strains used in this study are listed in Table 1. Enterococcal cultures were grown in Todd-Hewitt broth (THB; Difco, Detroit, MI, United States) at 37°C.

**TABLE 1:** Bacterial strains used in this study.

			**Multilocus**	**MIC (mg/L)*^a^***														
				
		**VRE**	**sequence**															
**Strain**	**Species**	**genotype**	**typing**															
				**VAN**	**TEC**	**LZD**	**AMP**	**GEN**	**STR**	**KAN**	**ERY**	**CHL**	**TET**	**MIN**	**CIP**	**CRO**	**CMZ**	**MEM**
AA708	*E. faecium*	*vanA/vanM*	78	256	8	2	512	>1024	>1024	>1024	>512	16	1	1	128	>1024	>1024	>1024
FA2-2/pELF1*^b^*	*E. faecalis*	*vanA/vanM*	–	1024	128	2	1	8	> 2048	>1024	>1024	4	1	2	1	32–64	512	8
BM4105RF/pELF1*^b^*	*E. faecium*	*vanA/vanM*	–	256	8	2	1	8	1024	>1024	>1024	4	1	2	3	1	64	8
ATCC9790RF/pELF1*^b^*	*E. hirae*	*vanA/vanM*	–	256	8	0.5	0.5	2	256	256	>1024	2	1	2	0.5	1	16	3
KT06RF/pELF1*^b^*	*E. casseliflavus*	*vanA/vanM/vanC*	–	256	16	1	0.5	3	512	>1024	>1024	4	1	2	1	16	64	1
FA2-2*^c^*	*E. faecalis*	–	–	2	1	2	1	8	64	64	0.5	4	1	2	2	512	512	16
BM4105RF*^c^*	*E. faecium*	–	–	1	1	2	1	8	32	1024	0.5	4	1	2	2	1	64	8
ATCC9790RF*^c^*	*E. hirae*	–	–	1	1	2	0.5	2	8	16	0.5	3	1	2	0.5	1	16	4
KT06RF*^c^*	*E. casseliflavus*	*vanC*	–	8	1	2	0.5	3	8	64	0.5	3	1	2	1	16	32	1
BM4105SS*^c^*	*E. faecium*	–	–	1	1	2	1	4	>2048	128	0.5	8	1	2	1	2	64	16
V583*^d^*	*E. faecalis*	*vanB*	–	256	1	2	0.5	>1024	64	>1024	>1024	8	1	2	0.5	128	512	4
ATCC29212*^d^*	*E. faecalis*	–	–	3	1	2	1	8	64	32	1	8	32	8	1	128	512	4
FA2-2/pMG2200	*E. faecalis*	*vanB*	–	64	1	2	2	16	64	128	0.5	8	1	–	1	1024	–	–

* ^*a*^VAN, vancomycin; TEC, teicoplanin; LZD, linezolid; AMP, ampicillin; GEN, gentamicin; STR, streptomycin; KAN, kanamycin; ERY, erythromycin; CHL, chloramphenicol; TET, tetracycline; MIN, minocycline;  CIP, ciprofloxacin; CRO, ceftriaxone; CMZ, cefmetazole; MEM, meropenem. ^*b*^Corresponding transconjugants obtained using filter mating with the donor AA708 strain. ^*c*^Used for PFGE and conjugation  experiments. ^*d*^Used as controls for drug susceptibility tests.*

### Drug Susceptibility Test

Minimum inhibitory concentrations (MICs) of antibiotics were determined using the agar dilution method. After each strain was grown overnight in Mueller-Hinton broth (MHB; Nissui, Tokyo, Japan), the cultures were diluted 1:100 with fresh broth. Approximately 5 × 10^5^ cells were spotted onto a series of Mueller-Hinton agar (Eiken, Tokyo, Japan) plate containing the appropriate test drugs. The plates were incubated at 37°C. The results were interpreted per the standards recommended by the Clinical and Laboratory Standards Institute guidelines^1^.

### Pulsed-Field Gel Electrophoresis (PFGE)

Pulsed-field gel electrophoresis was performed as described previously (Nomura et al., 2012). Agarose plugs (1%) containing embedded enterococci were treated with lysozyme (Roche Diagnostics K.K, Minneapolis, MN, United States) solution (10 mg/ml) at 37°C for 6 h, followed by treatment with proteinase K (Merck Millipore, Darmstadt, Germany) solution (60 mAnson U/ml) at 50°C for 48 h. After washing the plugs with wash buffer (20 mM Tris-HCl, pH 8.0; 50 mM EDTA), proteinase K was inhibited using phenylmethylsulfonyl fluoride (PMSF) solution (20 mM Tris-HCl, pH 8.0, 50 mM EDTA, and 1 mM PMSF). The plugs were digested at 37°C for 20 min with 5 U of S1 nuclease (Promega, Madison, WI, United States), and then subjected to PFGE using a CHEF-MAPPER (Bio-Rad, Richmond, CA, United States) according to the manufacturer’s instructions. To prepare non-S1 nuclease-treated plugs, the step for S1 nuclease treatment was omitted. The running condition was as follows: pulse from 1.0 to 12 s during 15 h at 6.0 V/cm at 4°C. After separation using PFGE, the DNA bands, corresponding to pELF1, were excised from the PFGE gel. To determine the physical map of pELF1, the excised plugs containing pELF1 DNAs were separately digested at 37°C for 24 h with 20 U of *Sal*I-HF (New England BioLabs, Ipswich, MA, United States), at 25°C for 24 h with 100 U of *Sma*I (New England BioLabs), at 37°C for 24 h with 150 U of *Eag*I-HF (New England BioLabs), and at 37°C for 24 h with 150 U of *Sac*I-HF (New England BioLabs). These DNA samples were then subjected to PFGE as described above.

### Multilocus Sequence Typing (MLST)

Multilocus sequence typing was performed as described previously (Homan et al., 2002). *atpA*, *ddl*, *gdh*, *purK*, *gyd*, *pstS*, and *adk* were sequenced, and the obtained sequence data were applied to *Enterococcus faecium* MLST databases^2^.

### Whole Genome Sequence (WGS) Analysis

AA708 was grown overnight in THB at 37°C. Bacterial cells were collected after centrifugation for 5 min at 12,000 × *g*, and the total DNA was prepared using Gentra Puregene yeast/bacteria kit (QIAGEN, Hilden, Germany). The DNA library for Illumina Miseq was prepared using the Nextera DNA Flex library preparation kit and Nextera DNA CD indexes (Illumina, San Diego, CA, United States) according to the manufacturer’s instructions and then sequenced as paired-end reads on an Illumina MiSeq platform using a MiSeq reagent kit v2 (300 cycles). On the other hand, the DNA library for nanopore MinIon was prepared using the SQK-LSK108 ligation sequencing kit according to the manufacturer’s protocol and then sequenced on a MinION Flow Cell (R9.4.1). WGS statistics is shown in Supplementary Table 1. After trimming the raw data, reads were assembled *de novo* using Unicycler (Wick et al., 2017). Minimap2 was used to visualize the short read alignments (Li, 2018). To obtain functional annotations, the assembled sequences were submitted to the DFAST Pipeline^3^ and RAST Server^4^ (Aziz et al., 2008; Brettin et al., 2015; Tanizawa et al., 2018).

### Sensitivity of pELF1 or pMG2200 to Exonuclease Treatment

S1 nuclease-untreated DNA bands excised from PFGE gels was used for exonuclease treatment. After washing the excised plugs with TE buffer, exonuclease treatment was performed using a modified procedure described previously (Kinashi and Shimaji-Murayama, 1991; Kalkus et al., 1993; Overhage et al., 2005; Rose and Fetzner, 2006; Dib et al., 2010). The DNA bands were digested with 100 U of exonuclease III (New England BioLabs) at 37°C for 1, 2, or 3 h. In addition, the DNA bands were digested with 10 U of lambda exonuclease (New England BioLabs) at 37°C for 15 h.

To remove the single-stranded DNA, 1 U mung bean nuclease (New England BioLabs) was used at 30°C for 0.5 or 1 h. S1 nuclease-treated pMG2200 was used as a control for linearized circular plasmid (Zheng et al., 2009).

### Sodium Dodecyl Sulfate (SDS)-PFGE and Retardation Assay

To investigate protein binding, enterococci embedded in plugs were treated with only lysozyme (non-proteinase K treatment) and used for SDS-PFGE. SDS-PFGE was performed as described previously (Stam et al., 1986; Kinashi and Shimaji-Murayama, 1991; Ravel et al., 1998; Shimizu et al., 2001). After adding SDS to both PFGE gel and running buffer to a final concentration of 0.2%, SDS-PFGE was performed using a CHEF-MAPPER (Bio-Rad) according to the manufacturer’s instructions. After separation using SDS-PFGE, non-proteinase K-treated DNA bands, corresponding to pELF1, were excised from the SDS-PFGE gel. These plugs, in which the intact proteins might be bound to pELF1, were used for the retardation assay. After washing thrice with Tris-EDTA (TE) buffer, restriction enzyme digestion was performed as described above. PFGE was performed after the plugs were inserted into the wells of a PFGE gel.

### Conjugation Assay and Transfer Frequency

Conjugation assays were performed as described previously (Dunny et al., 1978). AA708 was used as the donor strain. FA2-2 (*E. faecalis*), BM4105RF (*E. faecium*), KT06RF (*E. casseliflavus*), and ATCC9790RF (*E. hirae*) were used as the recipient strains. Briefly, donor and recipient strains were grown overnight in THB at 37°C. The overnight culture was diluted 50-fold in fresh THB, and then grown for 4 h at 37°C. Each 100 μl donor or recipient culture was mixed with 5 ml THB. The donor and recipient mixture were pressed through a 0.22 μm nitrocellulose filter (Merck Millipore, Darmstadt, Germany) using a syringe. After mating for 5 h on a THB plate, the nitrocellulose filter was washed with 1 ml THB via vortexing. The mating mixture was plated on a selective THB plate containing rifampicin (25 mg/L), fusidic acid (25 mg/L), and VAN (12 mg/L), and then incubated for 24 h. For the second conjugation assay, ATCC9790RF/pELF1 (*E. hirae*) and BM4105SS (*E. faecium*) were used as the donor and recipient strains, respectively. The selective THB plate for the second conjugation contained streptomycin (2048 mg/L), spectinomycin (256 mg/L), and VAN (5 mg/L). The transfer frequency was calculated as the number of transconjugants per donor cell. The values shown are the mean of three independent experiments with standard error.

## Results

### VanA/VanM-Type Vancomycin-Resistant *E. faecium*

During the evaluation of the multiplex PCR assay for VRE in our previous reports, we had identified a VanA/VanM-type vancomycin-resistant *E. faecium* strain from a > 60 year-old patient in Japan, which was identified as a VanA-type VRE strain (Nomura et al., 2018). This strain was detected from the stool sample. Unfortunately, we could not obtain detailed information regarding this patient. Similar to the results of a previous report (Sun H.L. et al., 2019), this strain was highly resistant to VAN (MIC = 256 mg/L) but showed intermediate resistance to teicoplanin (TEC) (MIC = 8 mg/L). Furthermore, the strain was also resistant to ampicillin, gentamicin, kanamycin, streptomycin, erythromycin, ciprofloxacin, cefmetazole, ceftriaxone, and meropenem (Table 1). MLST analysis revealed that this strain belonged to a ST78 lineage (allelic profile, 15-1-1-1-1-1-1) that has been reported to cause the hospital outbreak of VRE (Homan et al., 2002; Hsieh et al., 2010; Freitas et al., 2016).

### PFGE Analysis of the AA708 Strain

**FIGURE 1:**
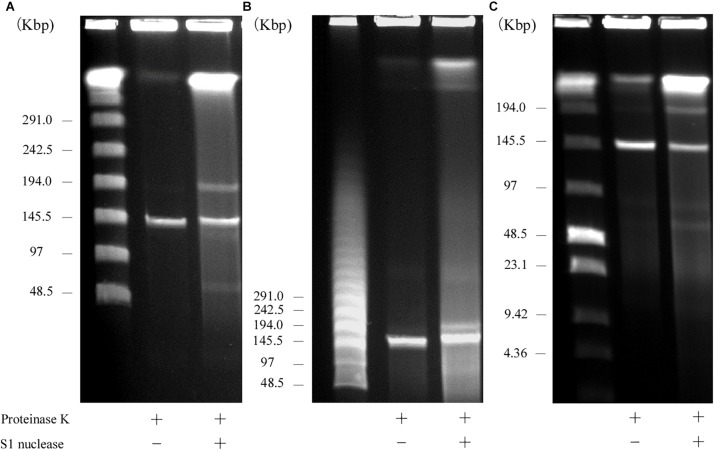
PFGE of S1 nuclease-treated or -untreated DNA of the AA708 strain in different running conditions. **(A)** The pulse time was from 1.0 to 23 s during 18.5 h of electrophoresis. Lanes: Lambda Ladder PFG Marker (NEB); S1 nuclease-untreated DNA; S1 nuclease-treated DNA. **(B)** The pulse time was from 50 to 90 s during 22 h of electrophoresis. Lanes: Lambda Ladder PFG Marker (NEB); S1 nuclease-untreated DNA; S1 nuclease-treated DNA. **(C)** The pulse time was from 1.0 to 12 s during 15 h of electrophoresis. Lanes: Lambda Ladder PFG Marker (NEB); S1 nuclease-untreated DNA; S1 nuclease-treated DNA.

S1 nuclease treatment is used to convert a supercoiled plasmid into linear molecules (Barton et al., 1995). To examine the plasmid content in AA708, S1 nuclease-treated DNA was subjected to PFGE. This analysis revealed that this strain harbors several plasmids (Figure 1). During the PFGE analysis, we noticed that one of these plasmids could be detected without linearization (Figure 1A), which was named pELF1 (enterococcal linear form plasmid). A previous report demonstrated that there was no difference in the electrophoretic mobility of linear plasmids treated with or without S1 nuclease (Cornell et al., 2018). To determine the pELF1 topology, we performed PFGEs under different running conditions. No difference in relative mobility is observed using different pulse time if the plasmid is linear (Shimizu et al., 2001; Dib et al., 2010). As expected, there was no change in the mobility when compared with the DNA size marker (Figures 1A–C).

### WGS Analysis of the AA708 Strain and Physical Map of pELF1

To determine the genetic structure, WGS using Illumina Miseq and Nanopore Minion was performed for the AA708 strain. Illumina data and Nanopore data were assembled *de novo* using Unicycler (Wick et al., 2017). The complete sequences of the genomic DNA of AA708, four circular plasmids, and one linear plasmid (pELF1) were obtained (Table 2).

**TABLE 2:** Detailed information regarding the plasmid contents of AA708.

		**Plasmid2**			
	**Plasmid1**	**(pELF1)**	**Plasmid3**	**Plasmid4**	**Plasmid5**
Size of each plasmid (bp)	193,900	143,316	63,548	6,326	6,173
Topology of plasmid*^a^*	Circular	Linear	Circular	Circular	Circular
Results of plasmid finder*^b^*	repUS15	No hit	rep17	No hit	rep11

*^*a*^WGS was performed using Illumina Miseq and Nanopore Minion and the data were assembled using Unicycler (Wick et al., 2017). ^*b*^*In silico* typing was performed using PlasmidFinder (https://cge.cbs.dtu.dk/services/PlasmidFinder/) (Jensen et al., 2010).*

**FIGURE 2:**
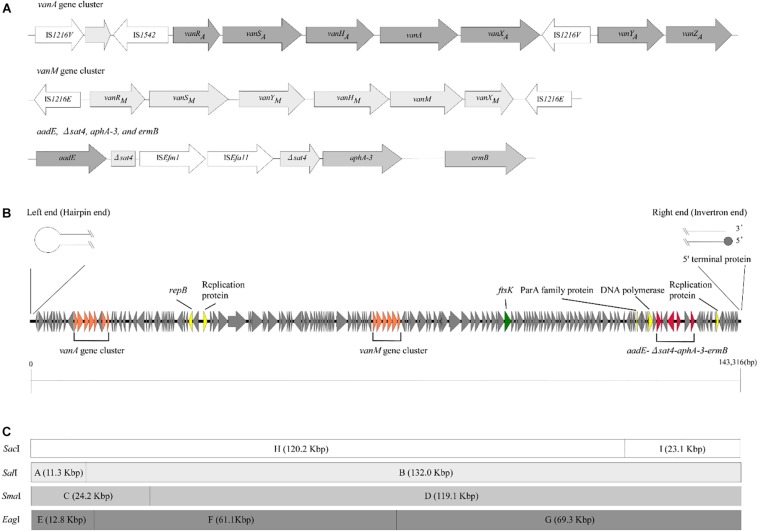
Genetic structure of vancomycin resistance, other antimicrobial drug resistance genes, and pELF1. **(A)** The genetic structure of vancomycin resistance genes and other antimicrobial drug resistance genes is shown. Elements IS*1216*V and IS*1542* were inserted in the Tn*1546*-like element harboring *vanA* gene cluster. In contrast, two IS*1216E* elements were located in the same direction at both ends of the *vanM* gene cluster. *aadE*, *sat4*, *aphA-3*, and *ermB* were located in pELF1 in this order. *sat4* was interrupted by IS*Efm1* and IS*Efa11*. **(B)** The schematic structure of pLFE1 is shown. The left terminal end formed a hairpin structure. On the other hand, the right end is presumed to be of the invertron type. Drug resistance genes as well as presumed replication machinery and transfer machinery genes are shown. The schematic structure was prepared using easyfig (Sullivan et al., 2011). **(C)** Restriction map of pELF1 (143.3 kb) is shown. A single *Sac*I, *Sal*I, and *Sma*I site each and two *Eag*I sites are present in pELF1. The fragments produced by these for restriction enzymes are denoted by letters.

To examine the *rep* sequence, we applied the sequence of pELF1 to PlasmidFinder 2.0; however no hit was found although the threshold for minimum percentage identity was 50% (Jensen et al., 2010; Carattoli et al., 2014) (Table 2). After annotation using the DFAST pipeline (Tanizawa et al., 2018), the *vanA* and *vanM* gene clusters were localized on pELF1. The *vanA* gene cluster was located on the mobile Tn*1546*-like element on pELF1. The nucleotide sequence of each *vanA* resistance gene was identical to that of BM4147, which showed high level of VAN resistance (Brisson-Noel et al., 1990; Arthur et al., 1993). Two copies of IS*1216*V and a IS*1542* were inserted in the Tn*1546*-like element (Figure 2A). This type of Tn*1546*-like element was a type II element, that was predominantly disseminated in hospitals in mainland China (Zheng et al., 2007). In contrast, the nucleotide sequence of the *vanM* gene cluster was identical to that of Efm-HS0661, which was the first VanM-type VRE isolated in Shanghai (Xu et al., 2010). Two IS*1216E* elements surrounded the *vanM* gene cluster were located in the same direction at both ends of the cluster (Figure 2A). This structure of the *vanM* gene cluster was identical to that of SRR22, which exhibited high level resistance to VAN (Sun L. et al., 2019). The *aadE*-*sat4*-*aphA-3* gene cluster, which encodes streptomycin, streptothricin, and kanamycin resistance, was also identified in pELF1 (Werner et al., 2001). This cluster contained IS*Efm1* and IS*Efa11* transposases, leading to the interruption of *sat4*. In addition, *ermB*, which encodes erythromycin resistance, was detected downstream of *aphA-3* (Lim et al., 2006) (Figure 2A). With the exception of *sat4*, all other genes were intact. Using the RAST annotation pipeline, the putative transfer-related genes *ftsK*, *parA*, and *repB* of the Rep_2 superfamily, which encode an initiation protein, were also identified on pELF1 (Bentley et al., 2004; Aziz et al., 2008; Brettin et al., 2015) (Figure 2B).

**FIGURE 3:**
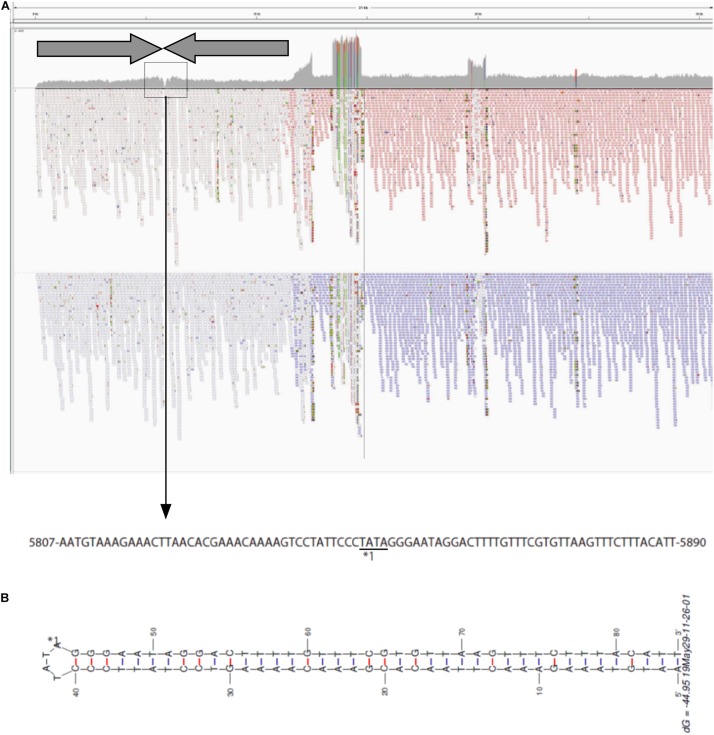
Visualization of the left-end sequence of pELF1. **(A)** Minimap2 was used to map short reads to the left-end sequence of pELF1 (Li, 2018). Dark arrows represented completely identical inverted direct repeats (IDR). These IDRs confronted each other at the region enclosed in a square. The nucleotide sequences in the square are shown below. **(B)** The structure of the left end of pELF1 was predicted using M. Zuker’s mfold server (http://unafold.rna.albany.edu/?q = mfold/DNA- Folding-Form) (Zuker, 2003). ^∗^1 indicates the sequence of the hairpin structure in the left end of pELF1.

To investigate the structure of the left end of pELF1, we carefully visualized the short read alignments to the prototype sequence at the left end of pELF1 (Figure 3). In the prototype sequence, approximately 5-kb inverted tandem repeat sequences, which were interspaced by four nucleotides (5′-TATA-3′), were detected (Figure 3A). This result was supported by the existence of several ultra-long reads (using the Nanopore system) encompassing these two 5-kb inverted repeat sequences. The coverage of short reads in this area was lower than that in other areas, and was the lowest at the center of these inverted tandem repeat sequences (Figure 3A). mfold analysis was used to investigate whether these inverted tandem repeat sequences formed a hairpin and double strand (Zuker, 2003). As expected, the mfold indicated that the left end formed a hairpin structure (Figure 3B). The sequence of the hairpin loop was 5′-TATA-3′. However, we could not detect the same hairpin structure in the right end.

**FIGURE 4:**
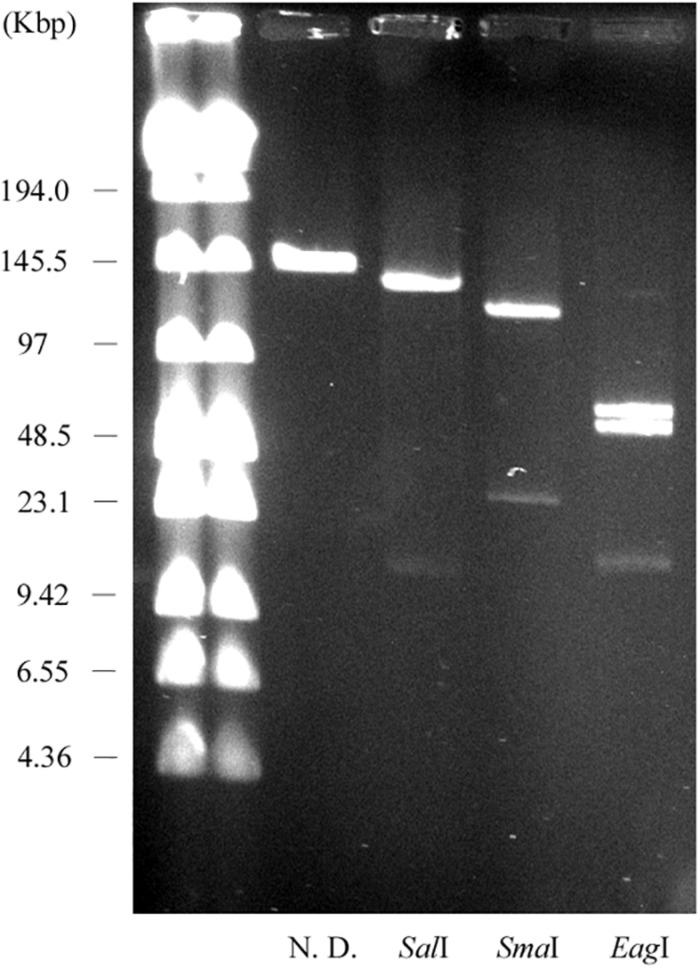
PFGE of the restriction fragments of pELF1 with proteinase K treatment. Lanes: Low Range PFG Marker; proteinase K-treatment only; proteinase K-treated *Sal*I digests; proteinase K-treated *Sma*I digests; proteinase K-treated *Eag*I digests. N. D., not digested.

We constructed physical maps of pELF1. Lysozyme and proteinase K-treated pELF1 was excised from the PFGE gel and digested with *Sal*I, *Sma*I, or *Eag*I, followed by PFGE (Figures 2C, 4). Based on the complete sequence data of pELF1, the number of cleavage sites for *Sal*I, *Sma*I, or *Eag*I was one, one, or two, respectively. As expected, each fragment number and the estimated sizes of the restriction fragments were consistent with those obtained from the complete sequence data (Figures 2C, 4).

### Analysis of Exonuclease Treatment for pELF1

**FIGURE 5:**
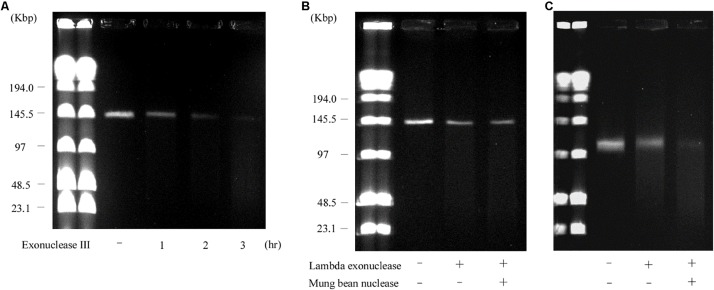
Sensitivity of pELF1 or linearized pMG2200 to exonuclease III or lambda exonuclease and mung bean nuclease treatment. **(A)** Sensitivity of pELF1 to exonuclease III treatment. Lanes: Lambder PFG Ladder; pELF1 without exonuclease III treatment; pELF1 with exonuclease III treatment (1 h); pELF1 with exonuclease III treatment (2 h); pELF1 with exonuclease III treatment (3 h). **(B)** Sensitivity of pELF1 or **(C)** linearized pMG2200 to lambda exonuclease and mung bean nuclease treatment. **(B)** Lanes: Lambder PFG Ladder; pELF1 without lambda exonuclease and mung bean nuclease treatment; pELF1 with lambda exonuclease treatment only; pELF1 with both lambda exonuclease and mung bean nuclease treatment. **(C)** Lanes; linearized pMG2200 without lambda exonuclease and mung bean nuclease treatment; pMG2200 with lambda exonuclease treatment only; linearized pMG2200 with both lambda exonuclease and mung bean nuclease treatment.

Contrary to the hairpin structure, the other type of linear plasmid is of the invertron type, which is characterized by termini attached to TPs (Sakaguchi, 1990; Hinnebusch and Tilly, 1993; Dib et al., 2015). Invertron types are more frequent and have been studied genetically. These TPs are covalently bound to the 5′-end and are involved in maintaining plasmid integrity and for telomere replication (Bao and Cohen, 2001; Yang et al., 2002; Tsai et al., 2008; Dib et al., 2015). As TPs are attached to the 5′ end of the linear plasmid, the latter are insensitive to 5′–3′ exonuclease (lambda exonuclease), but sensitive to 3′–5′ exonuclease (exonuclease III) (Kinashi and Shimaji-Murayama, 1991; Kalkus et al., 1993; Overhage et al., 2005; Rose and Fetzner, 2006; Dib et al., 2010). According to WGS analysis, the right end of pELF1 does not form a hairpin structure. We performed the exonuclease digestion analysis of pELF1 to investigate the structure of the right end. When both lysozyme and proteinase K-treated pELF1 was treated with exonuclease III, pELF1 was completely degraded (Figure 5A). In contrast, pELF1 did not degrade when treated with lambda exonuclease (Figure 5B). To remove single-stranded DNA, we used mung bean nuclease for lambda exonuclease-treated pELF1 (Kalkus et al., 1993; Rose and Fetzner, 2006); however, mung bean nuclease could not digest the exonuclease-treated plasmid (Figures 5B,C).

### SDS-PFGE and Retardation Gel Assay for pELF1

**FIGURE 6:**
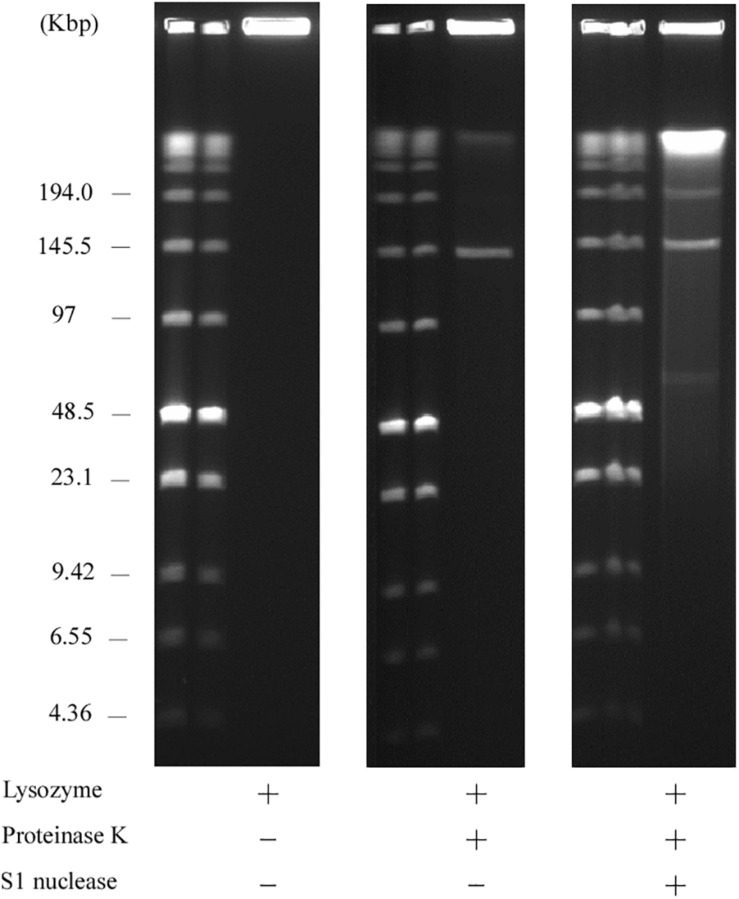
PFGE of the AA708 strain. Lanes: Low Range PFG Marker; AA708 without proteinase K treatment; AA708 with proteinase K treatment; AA708 with both proteinase K and S1 nuclease treatment.

**FIGURE 7:**
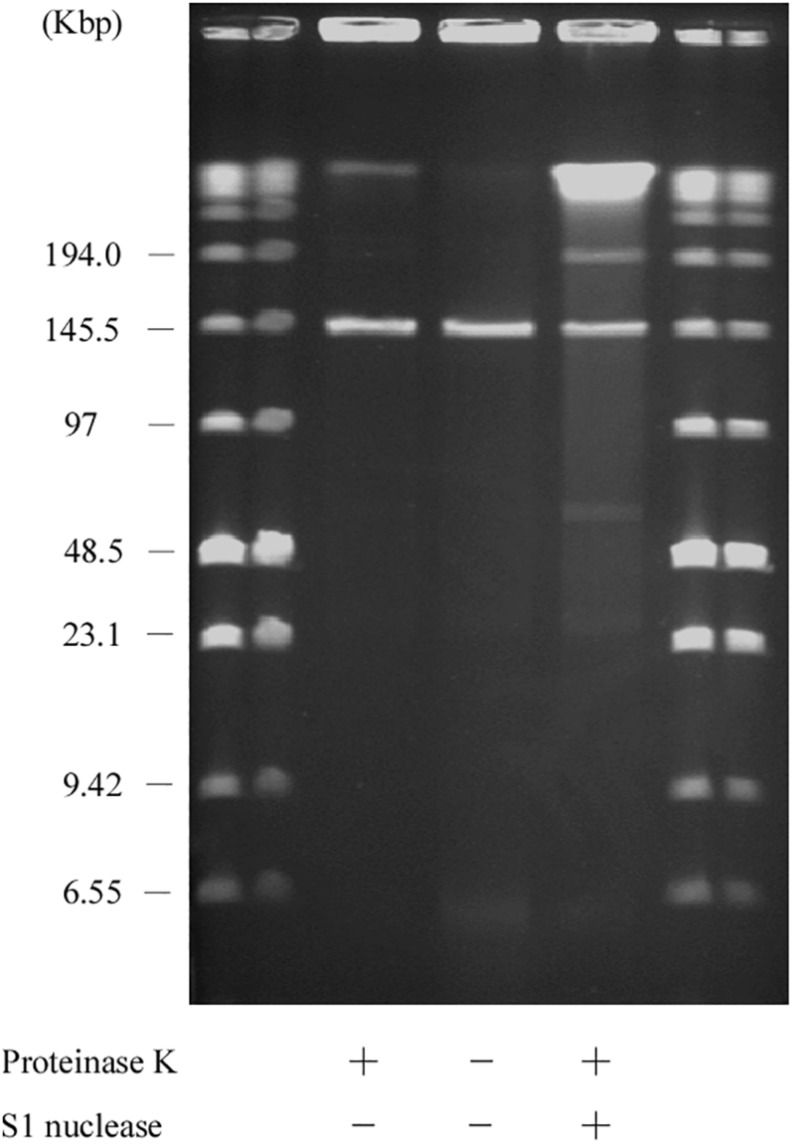
SDS-PFGE of the AA708 strain. Lanes: Low Range PFG Marker; AA708 with proteinase K treatment; AA708 without proteinase K treatment; AA708 with proteinase K and S1 nuclease treatment.

**FIGURE 8:**
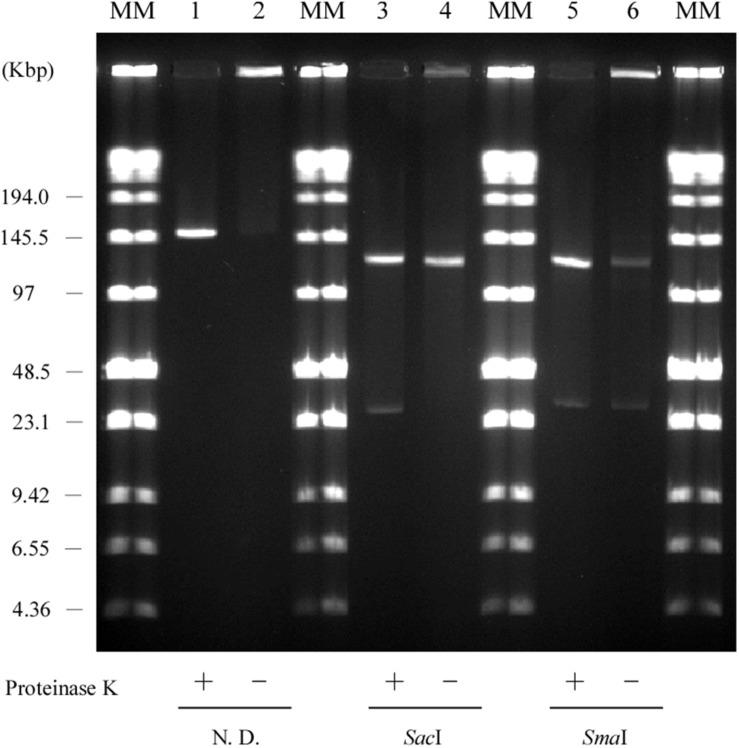
Retardation assay of pELF1 DNAs excised from PFGE or SDS-PFGE gels. DNA bands of pELF1 were excised from PFGE gel (proteinase K treatment +) or SDS-PFGE gel (proteinase K treatment –), and digested with *Sac*I (lanes 3 and 4), and *Sma*I (lanes 5 and 6). After digestion, the samples were subjected to PFGE. MM, Low Range PFG Marker; 1, pELF1 excised from PFGE gel; 2, pELF1 excised from SDS-PFGE gel; 3, *Sac*I-treated pELF1 excised from PFGE gel; 4, *Sac*I-treated pELF1 excised from SDS-PFGE gel; 5, *Sma*I-treated pELF1 excised from PFGE gel; 6, *Sma*I-treated pELF1 excised from SDS-PFGE gel. N. D.; not digested.

Usually, linear plasmids cannot move into a gel without proteinase K pretreatment as TPs are bound to these linear plasmids (Stam et al., 1986; Kinashi and Shimaji-Murayama, 1991; Ravel et al., 1998). Stam et al. (1986) reported that intact protein-linear plasmid complexes can migrate into the SDS-containing gel and running buffer as SDS unfolds the proteins (Ravel et al., 1998). In agreement with this, pELF1 without proteinase K treatment did not migrate, and remained at the origin of electrophoresis in PFGE (Figure 6). In contrast, pELF1 without proteinase K treatment moved as much as the proteinase K-treated pELF1 in SDS-PFGE (Figure 7). pELF1 excised from SDS-PFGE gel was believed to be attached to intact proteins, which resulted in its retention at the origin of electrophoresis in PFGE (Supplementary Figure 1). For the retardation assay, we used the proteinase K-untreated pELF1 excised from the SDS-PFGE gel. To confirm the retardation of the right end of pELF1, proteinase K-treated or untreated pELF1 were digested with *Sac*I and *Sma*I (Figure 8). In the lane for *Sac*I-digestion, a lower band (fragment I in Figure 2C) of the proteinase K-untreated pELF1 did not appear to move and was retained in the well (Figure 8, lane 4). In the lane for *Sma*I-digestion, the higher band (fragment D in Figure 2C) of proteinase K-untreated pELF1 appeared to be partially retained in the well, compared to the proteinase K-treated pELF1 (Figure 8, lane 6). These retained bands included the right end of pELF1, indicating that proteins might be attached to these DNAs (Figures 2B,C).

### Secondary Structure of the Right End of pELF1

In linear plasmids of *Streptomyces*, the terminal sequences (telomere) contain abundant palindromic sequences that can form secondary structures (Huang et al., 1998; Yang et al., 2017). These plasmids replicate bidirectionally from an internal origin, resulting in 3′ leading strand overhangs (Chang and Cohen, 1994). These single-stranded overhangs form hairpin structures that are involved in protection and replication of the linear plasmids (Huang et al., 1998; Mingyar et al., 2018). mfold was used to examine the right terminal sequences of pELF1 (Zuker, 2003). The analysis revealed that the 3′ leading strand overhangs of the right end of pELF1 harbored some palindromic sequences; however, this sequence was not consistent with the highly conserved sequence of *Streptomyces* (Mingyar et al., 2018) (Supplementary Figure 2). In contrast to typical *Streptomyces* telomeres, most hairpin loops in pELF1 consisted of four nucleotides. Only one GCGXAGC central motif, which was the recognition site of TPs, was detected in the palindromic sequence I of pELF1 (Kalkus et al., 1998; Bao and Cohen, 2003) (Supplementary Figure 2).

### Conjugation Assay

Horizontal transmission of VAN resistance genes via mobile genetic elements and clonal dissemination is a serious problem in clinical settings. In addition, conjugative linear plasmids of *Streptomyces* have already been reported (Chen et al., 1993; Hosted et al., 2004). This is a matter of concern as pELF1 harbors *vanA* and *vanM* gene clusters (Figures 2A,B). Conjugation experiments were performed to confirm the transferability of pELF1. We identified the transfer of pELF1 to FA2-2 (*E. faecalis*), BM4105RF (*E. faecium*), ATCC9790RF (*E. hirae*), and KT06RF (*E. casseliflavus*). pELF1 transferred at frequencies of 10^–5^, 10^–8^, 10^–8^, and 10^–3^ per donor cell to FA2-2, BM4105RF, ATCC9790RF, and KT06RF, respectively (Supplementary Table 2). pELF1 conferred VAN resistance and other drug resistances to these laboratory strains (Table 1). In addition, PFGE revealed that pELF1 of these transconjugants not treated with S1 nuclease can also migrate in the gel (Supplementary Figure 3). Moreover, we performed a secondary conjugation experiment with pELF1. ATCC9790RF/pELF1 and BM4105SS were used as the donor and recipient strains, respectively. pELF1 was transferred from the donor to the recipient strain, and pELF1 in the secondary transconjugant strain (BM4105SS/pELF1) also had a linear topology (Supplementary Figure 4A).

## Discussion

The transfer of a plasmid containing antimicrobial resistance genes often occurs in *Enterococcus* spp. and plays an important role in dissemination of drug resistance genes (Zheng et al., 2009; Yin et al., 2018). Previously, many linear plasmids have been reported, especially in *Streptomyces* spp.; however, linear plasmids have not been reported in Enterococci. In this report, we describe a linear plasmid harboring the *vanA* and *vanM* gene cluster. pELF1 harbored *vanA* and *vanM* gene clusters, showing high level of vancomycin resistance (Table 1).

Since the first isolation of VanM-type VRE in Shanghai in 2006, the incidence of VanM-type VRE has been increasing in clinical settings in China (Xu et al., 2010; Chen et al., 2015; Sun H.L. et al., 2019; Sun L. et al., 2019). The *vanM* gene cluster spread among unrelated enterococci via *in vivo* plasmid transfer (Sun L. et al., 2019). Recently, VRE harboring both *vanA* and *vanM* gene clusters were reported (Sun H.L. et al., 2019). The report described that the ST78 lineage is predominant among VanA/VanM-type vancomycin-resistant *E. faecium* in China (Chen et al., 2015; Sun L. et al., 2019). Our result was consistent with the observations of these reports. In addition, pELF1 can be transferred to other *Enterococcus* spp. *in vitro* and it encoded multiple antimicrobial drug resistance genes, including *aadE*, *aphA-3*, and *ermB* (Figures 2A,B, and Supplementary Figure 3). The drug-susceptibility test of transconjugants showed that pELF1 conferred STR, KM, and ERY resistance along with VAN resistance (Table 1). Taken together, this indicated that enterococci acquiring pELF1 showed multidrug resistance simultaneously and could survive under the selective pressure of these drugs. It also suggested that pELF1 in transconjugants such as *E. faecium*, *E. faecalis*, *E. casseliflavus*, and *E. hirae* had linear topology (Supplementary Figure 3). To examine the transferability of pELF1 in a transconjugant, we performed a secondary conjugation experiment between the ATCC9790RF/pELF1 (*E. casseliflavus*, donor) and BM4105SS (*E. faecium*, recipient) strains. This secondary conjugation was successful, and S1 PEGE results show that both the primary and secondary transconjugants did not harbor other plasmids except pELF1, suggesting that pELF1 was self-transmissible rather than mobilizable (Supplementary Figure 4). Transfer to other *Enterococcus* spp. and maintenance therein indicated that pELF1 harbored transfer-related and replication-related genes. In fact, pELF1 harbored regions of homology to the gene encoding cell division protein FtsK, which is possibly involved in the transfer of the *Streptomyces* linear plasmid pSLV45 (Hosted et al., 2004). *parA* of the partitioning protein (ParA) gene family, which is expected to contribute to low-copy linear plasmid segregation, and *repB* of the Rep_2 superfamily, encoding an initiation protein, were also detected (Bentley et al., 2004) (Figure 2B). In addition, the plasmid harbored several ORFs encoding hypothetical proteins (Figure 2B).

Some bacteriophages are known to be in linear form in extrachromosomes. For example, EF62phi, which was detected in *E. faecalis* 62, is linear (Brede et al., 2011). Many bacteriophages are found among enterococci, and the size of enterococcal phages can reach several 100 kb (Clewell et al., 2014). It is therefore extremely difficult to differentiate between linear plasmids and phage genomes based on genomic structure or size (Dib et al., 2015). Although we cannot exclude the possibility that pELF1 is an enterococcal phage, pELF1 did not harbor phage-related genes.

pELF1 harboring the *vanA* and *vanM* gene cluster migrated into the gel without S1 nuclease treatment, and the mobilities of the S1 nuclease-treated and untreated pELF1 did not differ (Figure 1), indicating that pELF1 was linear (Cornell et al., 2018). WGS analysis also suggested that pELF1 was a linear element, and the left end of pELF1 formed a hairpin structure. Minimap2 was used to visualize the short read alignments at the left end of pELF1 (Figure 3). We noticed that the coverage of short reads in the 5-kb inverted tandem repeat sequences was approximately half of that in the other area (Figure 3A). Minimap2 mapped short reads to a reference sequence only once (Li, 2018). If the sequence of the 5-kb inverted tandem repeats is correct, the coverage of this area did not decrease. Based on the result of mfold, these findings indicated that the 5-kb inverted tandem repeat sequences formed a hairpin structure (Figure 3B). In addition, the size of the complete sequence of pELF1 was consistent with the result of PFGE analysis and the physical map (Figures 1, 2C, 4 and Table 2).

The result of the exonuclease treatment revealed protein-binding to the plasmid, especially to the 5′ end of pELF1. Reports show that small peptides are still bound to the 5′ end of DNA after proteinase K treatment (Goshi et al., 2002). These peptides were presumed to prevent digestion by lambda exonuclease. Without proteinase K treatment, pELF1 could not migrate in PFGE gel; however, in SDS-PFGE gel, this could move at the same speed as proteinase K-treated pELF1 (Figure 7). This also suggested that the proteins were bound to pELF1. As the left end formed a hairpin structure, we hypothesized that the protein was bound to the 5′ end of the left end of pELF1. The results of the retardation gel assay were consistent with this result (Figure 8).

There are several reports regarding telomere sequences, TPs, and the replication mechanism of linear chromosomes, and plasmids, especially of *Streptomyces* spp. (Huang et al., 1998; Bao and Cohen, 2001; Yang et al., 2002, 2017; Huang et al., 2007; Zhang et al., 2009; Mingyar et al., 2018). The 3′ leading strand overhangs of *Streptomyces* linear DNA elements had six palindromic sequences. Telomere associated protein, which is essential for the replication of *Streptomyces* linear DNA elements, binds to palindrome II and III sites, leading to the recruitment of Tpg (Bao and Cohen, 2003). The Tap-Tpg complexes are involved in end patching using palindrome I as the template (Yang et al., 2017). These palindromic sequences, especially palindrome I, were highly conserved (Mingyar et al., 2018). In the 280 nucleotides of the right terminal sequence of pELF1, mfold detected 10 palindromic sequences forming hairpin structures; however, the sequence similarity was not high. We identified only one TP-binding motif in palindrome I of pELF1 (Kalkus et al., 1998; Bao and Cohen, 2003). Reports show that telomere sequence characteristics differ between pFiD188 of *Rhodococcus fascians* and *Streptomyces* linear plasmids (Francis et al., 2012). Based on the conjugation experiments, we considered that Tap and Tpg genes are strategically located on pELF1. To identify Tap and Tpg genes on pELF1, we used the Basic Local Alignment Search Tool (BLAST) and InterProScan (Jones et al., 2014). Some proteins have a helix-turn-helix domain suggesting DNA-binding functions; however, the size is not consistent with archetypal Tap/Tpg proteins (Kirby and Chen, 2011). In addition, among even *Streptomyces*, sequence similarity of Tap/Tpg might vary (Suzuki et al., 2008). Hence, it is not surprising that there might be Tap/Tpg and telomere sequence variation between the linear plasmid of *Enterococcus* spp. and other linear plasmids. pELF1 is therefore unique as it harbors two different terminal structures (Figure 2B). Further studies are underway to determine the TPs and telomere structures.

To our knowledge, this is the first report describing a linear plasmid in enterococci, which may be a clinical hazard because of its transferability to different enterococcal species carrying multidrug resistance genes including the *vanA* and *vanM* gene clusters. A limitation of this study is the lack of epidemiological analysis. Owing to the potential hazard in clinical settings, further study is required to examine the prevalence of linear plasmids in clinical enterococcal isolates and the corresponding transfer mechanism.

## Data Availability Statement

The dataset for this study (assembled sequence of AA708) can be found in the DNA Data Bank of Japan database (DDBJ) (https://www.ddbj.nig.ac.jp/) under the accession numbers LC495616 (pELF1), BLAC01000001 (Chromosome), BLAC01000002 (plasmid1), BLAC01000003 (plasmid3), BLAC01000004 (Plasmid4), and BLAC01000005 (plasmid5). The Illumina and Nanopore reads of AA708 strain have been deposited at DDBJ under the accession number DRA009065.

## Author Contributions

YH, TN, HH, KoT, GR, BZ, and HT conceived the project. KiT and TN collected the clinical materials. MT, KU, and YH conducted the NGS analysis. YH conducted the other laboratory work. All authors read and approved the final manuscript.

## Conflict of Interest

BZ was employed by the Institute of Clinical Pharmacology, Peking University First Hospital. KiT was employed by the company MIROKU Medical Laboratory, Inc. The remaining authors declare that the research was conducted in the absence of any commercial or financial relationships that could be construed as a potential conflict of interest.

## Footnotes

1 http://clsi.org/

2 https://pubmlst.org/efaecium/

3 https://dfast.nig.ac.jp/

4 http://rast.theseed.org/FIG/rast.cgi

## Abbreviations

MLST: multilocus sequence typing
ORFs: open reading frames
PFGE: pulsed-field gel electrophoresis
SDS: sodium dodecyl sulfate
Tap: telomere associated protein
TPs: terminal proteins
Tpg: terminal protein genes
VRE: vancomycin-resistant enterococci
WGS: whole genome sequence.
