# Supplementary material for: Novel Multidrug-Resistant Enterococcal Mobile Linear Plasmid pELF1 Encoding vanA and vanM Gene Clusters From a Japanese Vancomycin-Resistant Enterococci Isolate
Source: Front Microbiol. 2019 Nov 13;10:2568. doi: 10.3389/fmicb.2019.02568 (PMC6863802; doi:10.3389/fmicb.2019.02568)
Supplement: Supplementary file 1 [file Data_Sheet_1.docx]

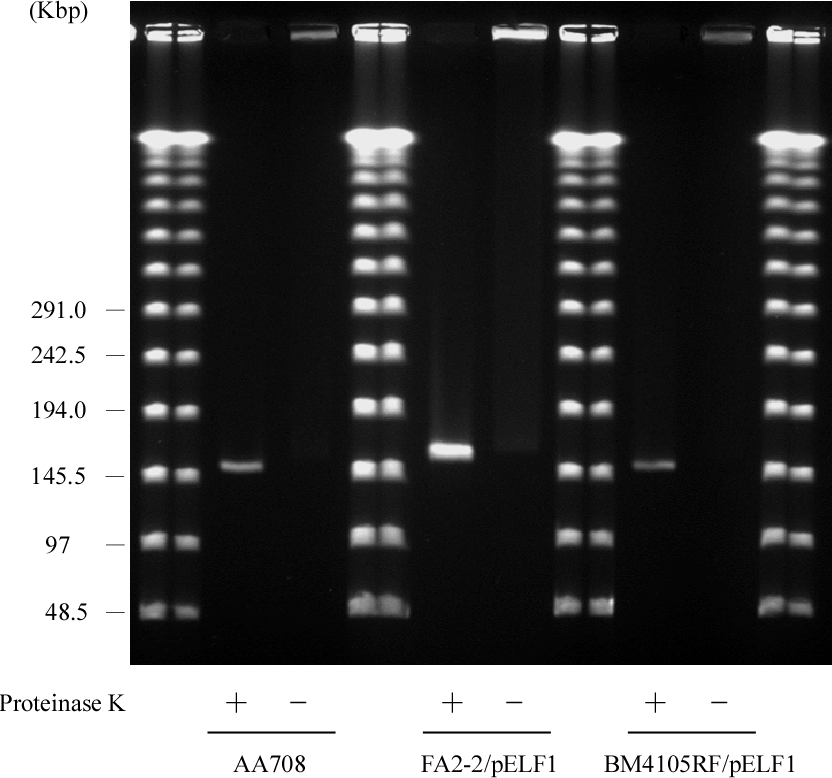


**Supplementary Figure 1. PFGE of pELF1 DNA excised from normal PFGE gel or SDS-PFGE gel.**

DNA bands of pELF1 were excised from normal PFGE gel (proteinase K treatment +) or SDS-PFGE gel (proteinase K treatment -), and subjected to normal PFGE without a restriction enzyme digestion. Lanes: Lambder PFG Ladder; pELF1 of AA708 excised from normal PFGE gel; pELF1 of AA708 excised from SDS-PFGE gel; pELF1 of FA2-2/pELF1 excised from normal PFGE gel; pELF1 of FA2-2/pELF1 excised from SDS-PFGE gel; pELF1 of BM4105RF/pELF1 excised from normal PFGE gel; pELF1 of BM4105RF/pELF1excised from SDS-PFGE gel.


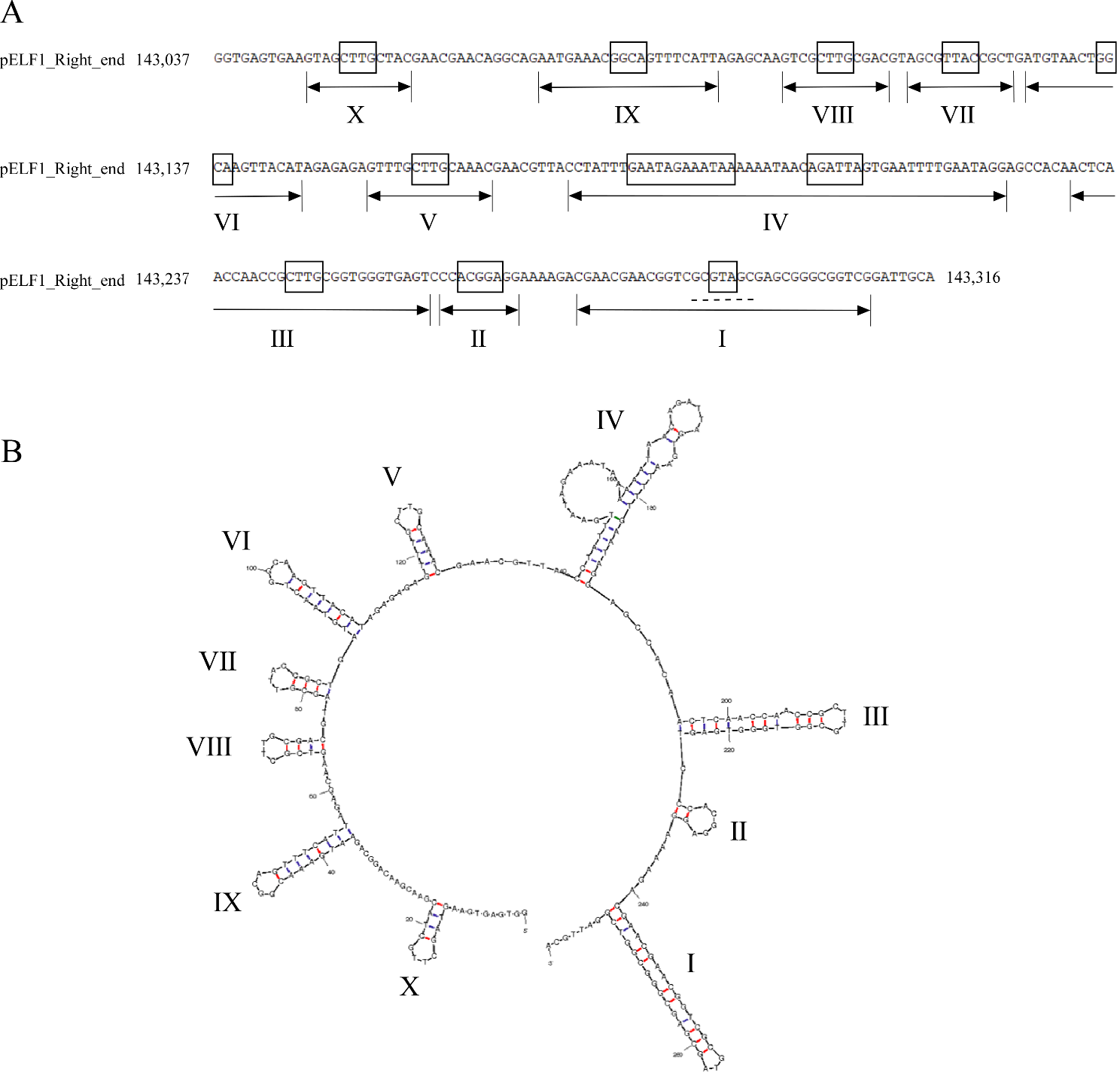


**Supplementary Figure 2. Terminal sequence and secondary structure of the right end of pELF1.**

(A) The 280 nt sequence of 3’ right end of the pELF1 was shown. Arrows and roman numerals indicated palindromic sequences. Boxes indicated loop sequences forming hairpin structures. Dashed line in Palindromic sequence I indicated the GCGXAGC central motif.

1. Predicted secondary structure of the 280 nt of 3’ right end of the pELF1 was determined by

mfold (ΔG = -53.57 kcal/mol).


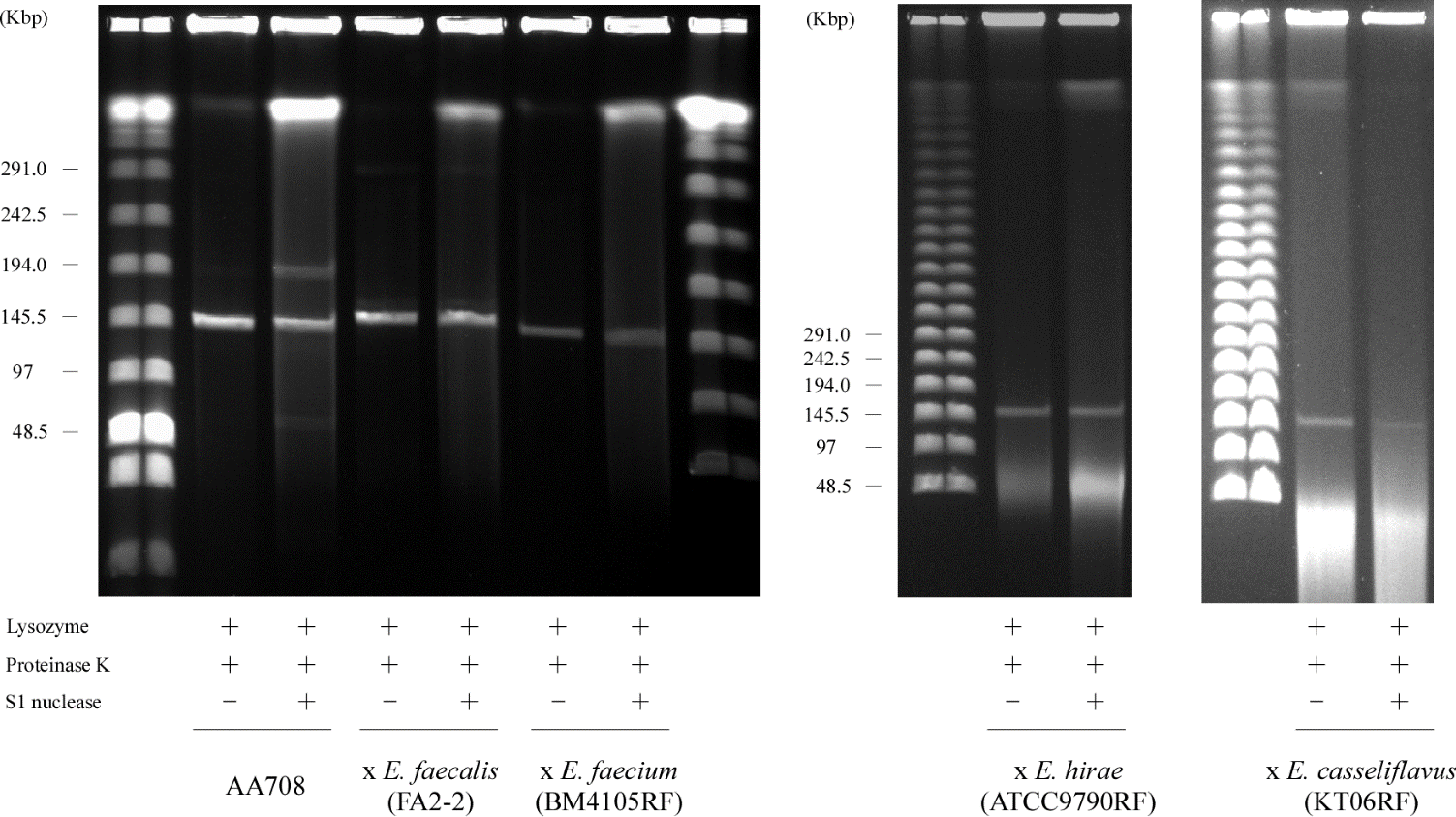


**Supple Fig. 3. PFGE of AA708 and its transconjugants.**

The plugs of AA708 strain or its transconjugants were used for normal PFGE analysis.

Lanes: Low Range PFG Marker; AA708 strain without S1 nuclease treatment; AA708 strain with S1 nuclease treatment; FA2-2/pELF1 without S1 nuclease treatment; FA2-2/pELF1 with S1 nuclease treatment; BM4105RF/pELF1 without S1 nuclease treatment; BM4105RF/pELF1 with S1 nuclease treatment; Low Range PFG Marker; Lambda PFG Ladder; ATCC9790RF/pELF1 without S1 nuclease treatment; ATCC9790RF/pELF1 with S1 nuclease treatment; Lambda PFG Ladder ; KT06RF/pELF1 without S1 nuclease treatment; KT06RF/pELF1 with S1 nuclease treatment. (The PFGE gel image of ATCC9790RF/pELF1 is as same as that in Supplementary Figure 4A.)


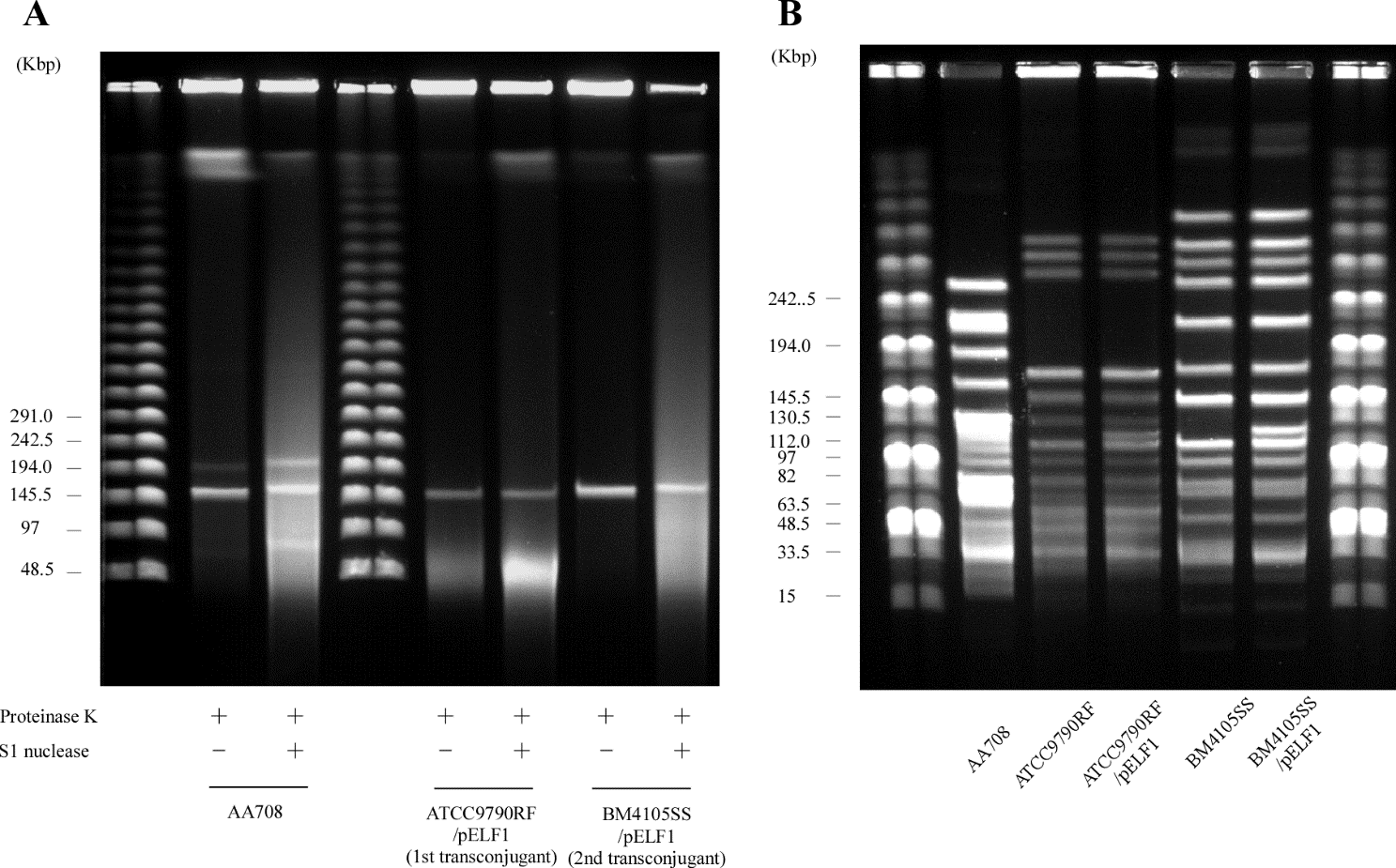


**Supple Fig. 4. PFGE of AA708 and its primary and secondary transconjugants.**

The plugs of AA708 strain or its transconjugants were digested with S1 nuclease (A) or *Sma*I (B), and used for normal PFGE analysis. (A) Lanes: Lambda PFG Ladder; AA708 strain without S1 nuclease treatment; AA708 strain with S1 nuclease treatment; Lambda PFG Ladder; ATCC9790RF/pELF1 without S1 nuclease treatment; ATCC9790RF/pELF1 with S1 nuclease treatment; BM4105SS/pELF1 without S1 nuclease treatment; BM4105SS/pELF1 with S1 nuclease treatment. (B) Lanes: Midrange PFG Ladder; AA708 strain; ATCC9790RF; ATCC9790RF/pELF1; BM4105SS; BM4105SS/pELF1. (The PFGE gel image of ATCC9790RF/pELF1 is as same as that in Supplementary Figure 3.)
